# Five Reasons Why Pediatric Settings Should Integrate the Play Specialist and Five Issues in Practice

**DOI:** 10.3389/fpsyg.2021.687292

**Published:** 2021-06-29

**Authors:** Giulia Perasso, Gloria Camurati, Elizabeth Morrin, Courtney Dill, Khatuna Dolidze, Tina Clegg, Ilaria Simonelli, Hang Yin Candy Lo, Andrea Magione-Standish, Bobbijo Pansier, Sandra Cabrita Gulyurtlu, Adam Garone, Hester Rippen

**Affiliations:** ^1^Department of Psychology, University of Milano-Bicocca, Milan, Italy; ^2^Porto dei Piccoli, Genoa, Italy; ^3^Children in Hospital Ireland, Dublin, Ireland; ^4^Children's Hospital of Philadelphia, Philadelphia, PA, United States; ^5^Georgian Association for the Care of Children's Health, Tiblisi, Georgia; ^6^Health Play Specialist Education Trust, Leicester, United Kingdom; ^7^Health Promoting Hospitals and Health Care Services – Children's and Adolescents' Task Force, Trento, Italy; ^8^Hong Kong Child Life Association, Hong Kong, China; ^9^Standish Foundation for Children, Austin, TX, United States; ^10^Starlight Children's Foundation UK, London, United Kingdom; ^11^Starlight Children's Foundation, Culver City, CA, United States; ^12^Stichting Kind en Ziekenhuis, Utrecht, Netherlands

**Keywords:** playing, children, adolescents, hospital, hospitalization, play specialists

## Introduction

Article 31 of the Convention on the Rights of the Child of the United Nations identifies play as a human right (Lundy, 2012) and the European Association for Children in Hospital (1988) lists play among the fundamental children's rights in healthcare (article 1–10). Playing is also a parameter to monitor the child's physical, emotional, cognitive, and executive development and well-being (Sutton-Smith, 1999; Koukourikos et al., 2015). Entering a medical setting exposes the child to many different risks for mental health (e.g., depression, withdrawal, regression, sleep problems, anxiety, hypochondria) because his/her familiar routine is disrupted (Chambers, 1993). In these cases, structured play-activities with a specialized professional can provide the child with a sense of continuity with the life before the illness (Romito et al., 2021) or with an imaginary escape from reality (Tanaka et al., 2010; Bukola and Paula, 2017).

In the 1920s, F. Nightingale and F. Erikson were the first nurses intuiting the importance of systemizing playing sessions to ameliorate children's hospitalization experience and adherence to medical procedures (Frauman and Gilman, 1989; Francischinelli et al., 2012). Then, the books “Working with Children in Hospital” (Plank, 1962), “Children in the Hospital” (Bergmann, 1965), “Play in Hospital” (Harvey and Hales-Tooke, 1972) highlighted that introducing a specialist in play-activities in the hospital was fundamental for the child's psychosocial well-being. By the same token, Brooks (1970) remarked that the “Play Lady” should not be considered a recreational figure for hospitalized children but a psycho-pedagogical intervener that supports the child when he/she is coping with the illness. As Rubin (1992) points out, a large body of synonyms (e.g., play lady, puppet lady, playing checkers, playing teacher, recreational therapist) was used between the '60 and the '80 to describe the same role. Nowadays, there are still many synonyms describing these professionals (e.g., Healthcare Play Specialist, Certified Child Life Specialist, Child Play Specialist, Medic Pedagogic Healthcare worker), and the need for creating scientific consensus around this role is urgent. As emerged from 29th January 2021 Virtual Round Table “Playing in the Hospital,” most of the international stakeholders indicated the term “Play Specialist” (PS) as an encompassing worldwide macro-label to describe this professionalism (Porto dei Piccoli, 2021). The PS differs from the play-therapist since play therapy is a counseling technique used in psychoanalytic psychotherapy (Leblanc and Ritchie, 2001). All over the world, hospitals, trusts, and charities often promote the PS in the pediatric care settings. No-profit organization are crucial to promote the PS in countries where the role is not integrated yet in the healthcare system. The lack of international guidelines for the PS practice leads these professionals to theoretical and operative fragmentation, challenges, and issues that Covid-19 pandemic is further stressing out. The aim of the present paper is promoting knowledge about the PS by defining the professionalism, analyzing the obstacles that hinders the PS practice, and emphasizing the reasons why promoting the PS in pediatric care settings (e.g., hospitals, home-based care).

## Who Is the Play Specialist? What Does the Play Specialist Do?

Notwithstanding countries terminological differences (e.g., in the UK the PS is named Healthcare Play Specialist, in the USA and Canada is named Certified Child Life Specialist), the PS can be described in the light of a common body of practice. Firstly, all over the world, becoming a PS requires a specific training accessible with a bachelor's degree in psychological or pedagogical sciences as a prerequisite (Harvey, 1984; Lookabaugh and Ballard, 2018). In several countries (e.g., Netherlands, UK, US) the Play Specialist is an official education degree, in others (e.g., Italy) the training is organized and financially supported by trust and charities, with discretion in the duration and total hours. Generally, the PS training focuses on the child development's milestones (e.g., physical, cognitive, communicative, emotion regulation, social skills maturation) from a medical, psychological, and pedagogic point of view, to enable PS to provide children with age-specific and diagnoses-specific play activities (Beickert and Mora, 2017). Completing a certified training is crucial because it predicts the use of research-based strategies by the PS to work with the child (Bandstra et al., 2008). Once trained, the PS can support children with various play techniques. Among a wide range of actions, the most practiced ones are the normative play and medical play (Burns-Nader and Hernandez-Reif, 2016). The normative play encompasses all the play activities that the child would experience at home. It conveys the message that the child can play and be creative in the hospital as he/she does in well-known places. On the other hand, the medical play helps the child to learn about health and illness and to familiarize herself/himself with the hospital context, aiming at reducing the child's anxiety toward medical procedures. According to Barry (2008), such activities can also occur outside the hospital ward by organizing house-visits and experiential weekends. Such experiential occasions help children with specific chronic conditions (e.g., diabetes) to increase their health-related self-efficacy outside their comfort-zone.

A few studies have attempted to profile the PS. In the US, Lookabaugh and Ballard (2018) survey on the Child Life Specialists reports that most PS work in hospitals (93% of the respondents, n = 147). Bottino et al. (2019) add that the Child Life Specialists are mostly females, in their thirties, with 88% respondents (total *n* = 110) working to ameliorate children's coping, family perception of support, children collaboration in medical procedures. Similar surveys were conducted in Japan (Tanaka et al., 2010) and New Zeland (Goh et al., 2019), while Europe still lacks survey-evidence about PS. Much needs to be done to profile the true nature and prevalence of this profession across the globe to build a common ground for practice.

## Five Challenges in Practicing the PS Profession and Suggested Solutions

Both in countries where the healthcare system does not incorporate the PS as well as in countries where the PS professionalism is acknowledged, a variety of issues arise. The following challenges are analyzed in the light of possible solutions. (i) Creating a common ground for research: the proliferation of different labels, and operative strategies hindered creating an evidence-based framework for PS professionalism. A standardized data collection system should be implemented (Goh et al., 2019). Different-countries stakeholders should build methodological consensus addressing at: (a) facilitating information sharing with the medical staff and (b) corroborating the reputation of this professional with empirical evidence. (ii) Implementing specific intervention strategies: given the link between practice heterogeneity and research fragmentation, international stakeholders should isolate the core-PS activities (i.e., normative play and medical play) from supplementary actions (i.e., educational video gaming and pet-therapy) (Kaminski et al., 2002; Jurdi et al., 2018) to analyze the effect of the intervention. Plus, age-specific, and diagnosis-specific evidence-based intervention protocols should be built, tested, and shared across countries. (iii) Health Providers need to recognize PS as an essential part of the team: PS intervention is complementary to the doctors' and nurses' work, and it aims at explaining to children the procedure they apply and make them comfortable in the interactions with the medical staff (Metzger et al., 2013; Beickert and Mora, 2017). The hospital staff may be confused by the lack of acknowledgment of the PS in the healthcare field. They could consider the PS a ward volunteer, and sometimes the nurses and doctors may play with children themselves (e.g., especially before stressing medical procedures), without having the PS background and a structured play program (Tanaka et al., 2010; Kihara and Yamamoto, 2018). Managing misperceptions and improper role-overlapping should be a priority to enhance the alliance between PS and hospital staff. (iv) Policy-makers, commissioners, and funders should invest in PS as part of mainstream health care delivery: Charities, trusts, and several stakeholders worldwide financially support the PS and children's rights (Simonelli et al., 2014). Gaining more visibility through an international consortium may increase the attention paid to this role in national and international healthcare systems. Joined actions to concur in international calls for funding may also result in a crucial strategy to increase funds. (v) Supporting hospitals during Covid-19 pandemics: During the Covid-19 pandemic, several countries' governments blocked access to pediatric hospitals to external professionals from non-governmental associations. In Italy, such restriction led many families to be deprived of PS support or to rely on the PS online intervention (Perasso et al., 2020). Given the vital role of PS in children's healthcare, pandemics crisis could constitute an opportunity to incorporate the PS in the healthcare system. In fact, it has never been more urgent to make hospitals friendly and safe again in children and families' perception. Stakeholders, trusts, and charities should dialogue with policy makers to regulate the PS access to hospital (e.g., discussing Covid-19 testing and safety equipment), and to engage the PS in future children's anti-Covid vaccination campaigns.

## Five Reasons Why Medical Settings Should Integrate the Play Specialist

Given that in many countries the PS is not yet integrated in the medical settings, the need for further recognition for this role is pending to build a global common ground for the PS. In line with purpose, the present paragraph points out the research findings demonstrating that the PS intervention positively impacts both the child and the medical setting. Five main reasons based on evidence are provided. (i) Improving the child well-being: research demonstrates that the PS intervention effectively improves coping strategies and reduces anxiety and stress levels among hospitalized children (Gill, 2010; Moore et al., 2015; Li et al., 2016; Ullan and Belver, 2019). Evidence from a survey administered to doctors and nurses reveals that having a PS in the hospital ward, who effectively provides the child with distraction techniques, helps to ensure that the pediatric patient is relaxed during medical procedures (Tanaka et al., 2010; Bukola and Paula, 2017). (ii) The child needs less sedation for pain management: literature shows that the PS intervention improves the child's pain management strategies (i.e., behavioral, physical, cognitive, and complementary), replacing the need for pharmacological protocols and sedation (Bandstra et al., 2008). Evidence from the UK (Gulyurtlu et al., 2020), documenting over 1,000 responses from health professionals on the impact of play-specialist intervention, demonstrates that distraction play techniques can reduce children's feelings of pain associated with hospital treatment. (iii) The child is more adherent to the medical treatment: The PS intervention (e.g., distraction techniques) can decrease negative emotions, resulting in treatment compliance by the pediatric patient (Gill, 2010; Taddio and McMurtry, 2015). When a child is hospitalized, she/he also experiences power imbalance about health-related decisions as adults choose on behalf of her/him (Bricher, 2000), leading her/him to opposing reactions. Play can empower the child in the medical setting (Li et al., 2016) as the PS represents a crucial mediator to advocate the child's right in the medical setting by informing her/him about adult's choices, corroborating the child's sense of responsibility on health-related issues and enhancing the compliance toward treatments. (iv) Play is an essential part of palliative care: the PS support is crucial in palliative care to provide children with compensatory experiences, to create supportive bonds for the family system, and to psychologically prepare parents for the eventuality of loss (Basak et al., 2019). (v) Savings for the hospitals: A single case study by Metzger et al. (2013) points out that the PS intervention effectively reduced a 8-year-old patient's anxiety before MRI-examination. After the PS intervention, the radiologist did not evaluate the anesthesia necessary before MRI with two positive consequences: the child was spared from anesthesia, and the hospital saved the anesthesia cost. Grissom et al. (2016) reported a similar finding on a sample of n = 116 children, aging between 5 and 12, affected by nervous system diseases. It is also worth noticing that specific diagnoses and symptoms (e.g., attention deficit and hyperactivity disorder, autism spectrum disorder, intellectual disabilities, borderline personality traits) in children and adolescents are particularly challenging for doctors and nurses (Jaunay et al., 2006; Scarpinato et al., 2010; Brinkman et al., 2011). Having a PS effectively providing tools to increase emotion and behavioral regulation in a wide range of diagnosis among pediatric patients may save hospital personnel's time and spare doctors and nurses from physical efforts and psychological stress.

## Conclusion

The paper aims to sensitize the scientific community about the importance of play-specialist's intervention for ill children in medical settings, and to raise awareness in policy-makers for officially integrating the PS in the healthcare field. Internationally, stakeholders, trusts, and charities are pointing out that the PS is a vital professionalism to advocate the psychosocial well-being of the pediatric patients and their families.

## Author Contributions

GP written the paper. GC, EM, CD, KD, TC, IS, HL, AM-S, BP, SG, AG, and HR contributed to the conception of the paper. All authors contributed to the article and approved the submitted version.

## Conflict of Interest

The authors declare that the research was conducted in the absence of any commercial or financial relationships that could be construed as a potential conflict of interest.
